# Machine learning-driven blood biomarker profiling and EGCG intervention in fetal alcohol spectrum disorder

**DOI:** 10.1016/j.ijchp.2025.100620

**Published:** 2025-09-04

**Authors:** Anna Ramos-Triguero, Elisabet Navarro-Tapia, Melina Vieiros, Leopoldo Martínez, Óscar García-Algar, Vicente Andreu-Fernández

**Affiliations:** a Grup de Recerca Infancia i Entorn (GRIE), Institut d'investigacions Biomèdiques August Pi i Sunyer (IDIBAPS), Barcelona, Spain; b Department de Cirurgia i Especialitats Mèdico-Quirúrgiques, Universitat de Barcelona, Barcelona, Spain; c Redes de Investigación Cooperativa Orientadas a Resultados en Salud (RICORS), Instituto de Salud Carlos III (ISCIII), Madrid, Spain; d Institute for Biomedical Research La Paz (IdiPaz), Madrid, Spain; e Faculty of Health Sciences, Valencian International University (VIU), Valencia, Spain; f Department of Pediatric Surgery, Hospital Universitario La Paz, Madrid, Spain; g Department of Neonatology, Hospital Clínic-Maternitat, ICGON, BCNatal, Barcelona, Spain; h Biosanitary Research Institute, Valencian International University (VIU), Valencia, Spain

**Keywords:** Fetal alcohol spectrum disorder (FASD), Blood-based biomarkers, Epigallocatechin gallate (EGCG), Machine learning, Artificial intelligence, Brain health, Diagnosis

## Abstract

Fetal alcohol spectrum disorder (FASD) is a complex neurodevelopmental condition caused by prenatal alcohol exposure (PAE), often underdiagnosed due to heterogeneous symptoms and diagnostic challenges. This study aimed to identify serum-based biomarkers for early FASD diagnosis and assess the potential of epigallocatechin gallate (EGCG), a natural antioxidant found in green tea, in modulating markers related to FASD. Luminex immunoassays were employed to analyze serum samples from FASD patients, identifying seven predictive biomarkers involved in neuroinflammation and immune dysregulation: IL-10, IFNγ, CCL2, NGFβ, IL-1β, CX3CL1, and CXCL16. These biomarkers reflect key disruptions in brain health, particularly in neuroinflammation, which contributes to the cognitive, behavioral, and mental health challenges frequently observed in FASD patients, including memory deficits, attention problems, and emotional dysregulation. To enhance diagnostic precision, machine learning (ML) models were trained on these biomarker datasets, with Random Forest (RF) achieving the highest accuracy (0.89), sensitivity (0.92), specificity (0.83), and ROC AUC (0.88). Additionally, an open-label pilot study in children diagnosed with FASD showed significant restoration of the levels of IFNy, CX3CL1, IL-1β, IL-10, and NGFβ after 12 months of EGCG treatment, suggesting its potential role in mitigating neuroinflammatory responses and promoting neurogenesis. These findings underscore the value of integrating serum biomarkers with ML-driven approaches to advance FASD diagnostics, while also identifying EGCG as a promising intervention for neurodevelopmental and mental health impairments associated with the disorder.

## Introduction

Fetal alcohol spectrum disorder (FASD) is a multifaceted neurodevelopmental disorder associated with prenatal alcohol consumption (PAE) (Chung et al., 2021), which includes Fetal Alcohol Syndrome (FAS), partial FAS (pFAS), alcohol-related neurodevelopmental disorder (ARND), and alcohol-related birth defects (ARBD) (Hoyme et al., 2016). Globally, FASD affects approximately 7.7 per 1000 individuals, with prevalence in Europe reaching up to 19.8 per 1000 (Lange et al., 2017a). Eastern European orphanages have reported rates between 15% and 68% (Popova et al., 2014), and it is known that at least half of the children adopted from Eastern European countries exhibit symptoms linked to FASD (Colom et al., 2021; Landgren et al., 2010). In Western European countries and the United States, 1–5% of school-aged children may be affected by FASD (May et al., 2009, 2018). Despite its high prevalence, FASD remains underdiagnosed due to the variability of symptoms and overlap with other conditions such as attention deficit hyperactivity disorder (ADHD).

Individuals diagnosed with FASD experience lifelong neurocognitive impairments and social challenges. These include deficits in memory, attention, adaptive functioning, and abstract reasoning (Champagne et al., 2023; Kelly et al., 2000; Lange et al., 2017b). Secondary disabilities arise from interactions between primary impairments and the environment, contributing to difficulties in education, employment, mental health, and social relationships (Lowry, 1997) leading to academic failure, low self-esteem, unstable housing, depression, and legal problems (Leenaars et al., 2012; Navarro et al., 2011; Pei et al., 2011; Popova et al., 2016). Therefore, early diagnosis is essential to facilitate targeted interventions, improve quality of life, and reduce long-term complications.

Current diagnostic criteria for FASD encompass PAE history, characteristic facial features, growth impairments, and neurodevelopmental deficits (Hoyme et al., 2016). The Institute of Medicine (IOM) guidelines include assessments of craniofacial anomalies, growth retardation, and neurodevelopmental disorders (Hoyme et al., 2016). However, challenges such as unreliable maternal alcohol histories, changes in custody, and the absence of clear physical features hinder accurate diagnoses. As a result, many cases remain undiagnosed or are identified too late, limiting access to early interventions and support for academic difficulties, employment instability, mental health, drug use, legal problems, and suicide attempts (Fast & Conry, 2009; Jańczewska et al., 2019; Popova et al., 2016).

A key consequence of PAE is the alteration of cytokine and chemokine pathways, such as CCL2 (Chang et al., 2018; Pascual et al., 2017; Zhang & Luo, 2019), CX3CL1 (Bachstetter et al., 2011; Cardona et al., 2006; Pascual et al., 2017; Roberson et al., 2011), and CXCL16 (Juárez-Rodríguez et al., 2020; Lepore et al., 2018; Rosito et al., 2012, 2014). The imbalance of these molecules has been associated with alcohol-induced neuroinflammation in the developing central nervous system (CNS), as well as in various neurodevelopmental conditions, such as Alzheimer (Domingues et al., 2017; Sanford & McEwan, 2022), Parkinson (Liu et al., 2019) and ADHD (Dunn et al., 2019). PAE also triggers impaired activation of microglia and astrocytes (Kane & Drew, 2021; Lawrimore et al., 2019; Lord et al., 2022), releasing pro-inflammatory cytokines such as IL-1β, IL-6, TNF, COX2, and nitric oxide, as well as a reduction in anti-inflammatory factors such as IL-10 (Lobo-Silva et al., 2016; Milligan & Watkins, 2009; Pascual et al., 2017; Roberson et al., 2012; Saijo & Glass, 2011). Additionally, there is an exacerbated leukocyte migration to the CNS due to an altered expression of adhesion molecules and remodeling proteins such as MMP-10 and VCAM-1 (DeVito & Stone, 2001; Noor & Milligan, 2018).

In addition to the molecules mentioned above, there is a subset of neuronal biomarkers, including Enolase-2 (a marker of cell maturation in nerve tissue) (Ledig et al., 1990), NGF (neuronal plasticity marker) (Boschen & Klintsova, 2017; Ceccanti et al., 2012) as well as s100b (neural differentiation marker) (Eriksen et al., 2002), that have been identified as potential indicators of neuroinflammation and neuronal damage. All these biomarkers could be used, after clinical validation, as potential candidates to facilitate the diagnosis of this syndrome. Nevertheless, studies specifically targeting serum biomarkers in human FASD patients remain limited.

The search for novel strategies and methods for early diagnosis is one of the most promising fields of research in FASD. In this context, emerging technologies, such as machine learning (ML), offer promising avenues for improving diagnostic accuracy and efficiency (Rodrigues et al., 2023). ML algorithms have demonstrated impressive capabilities in analyzing complex datasets and extracting meaningful patterns in other diseases such as cancer, Alzheimer, ASD, and ADHD (Bahathiq et al., 2022; Ehrig et al., 2023; Eslami et al., 2021). For instance, ML has shown great promise in predicting the onset of Alzheimer's disease, specifically in identifying individuals at risk of significant cognitive decline, thereby facilitating their inclusion in clinical trials (Ezzati et al., 2020). In recent years, research exploring the potential use of ML algorithms for early diagnosing FASD has shown promising results (Suttie et al., 2024). Goh et al. trained their model using CBCL scales, IQ, and physical examination, obtaining a sensitivity of 64%−81% and specificity of 78%−80% (Goh et al., 2016). Furthermore, Lussier *et al*. used methylation signatures for FASD classification (Lussier et al., 2018). Using facial recognition datasets, Blanck-Lubarsch et al. formulated an automated classification algorithm with 3D facial scans (Blanck-Lubarsch et al., 2022). However, further research into the application of machine learning algorithms is essential to improve early diagnosis and enhance the quality of life for individuals. Additionally, integrating serum biomarkers into ML models could further increase diagnostic accuracy and facilitate earlier detection of FASD.

In addition to improved diagnostic accuracy, effective treatments are needed to combat the harmful effects of PAE. Epigallocatechin gallate (EGCG), a catechin found in green tea, has been investigated as a potential treatment for several health conditions, including cancer, inflammation, diabetes, and cardiovascular diseases (Chu et al., 2017). Despite some molecular mechanisms remain unclear, EGCG has been shown to attenuate oxidative stress (Saffari & Sadrzadeh, 2004), protect against neuroinflammation, boost the immune system (Levites et al., 2003; Long et al., 2010), and provide neuroprotective benefits in neurodegenerative diseases (de la Torre et al., 2016). In addition, EGCG has received increasing attention for its role in neural plasticity and its interaction with key proteins such as DYRK1A (De la Torre et al., 2014; de la Torre et al., 2016). Previous studies have examined the protective effects of EGCG in FASD, including oxidative stress, cardiac biomarkers, neuronal maturation, astrocyte differentiation, and neuronal plasticity (Almeida-Toledano et al., 2021; Andreu-Fernández et al., 2023).

The present study has analyzed the serum protein profile in a cohort of children diagnosed with FASD to identify specific biomarkers associated with this syndrome for diagnostic purposes. ML algorithms were used to develop a predictive model based on the serum protein profile for the early diagnosis of FASD. In addition, the effect of 12-month treatment with the antioxidant EGCG on these biomarkers was studied to determine the potential of this natural antioxidant as a therapeutic tool for FASD.

## Material and methods

### Study design and participant information

This is an open-label, pre-post intervention, non-randomized quasi-experimental study. The study cohort comprised 30 children without a diagnosis of FASD (control group) and 60 patients diagnosed with FASD, all of them coming from adoptions from Eastern European countries (EEC). The subset of 60 FASD patients received a nutritional supplement (FontUp®), a therapeutic candidate known for its potential to enhance cognitive performance (de la Torre et al., 2016). FontUp® is a soluble commercial preparation in a sachet format containing 94% EGCG (Supplementary Table S1). The selection of FontUp® for this study was based on several key factors. First, its palatable taste makes it easier for children to consume, overcoming a common challenge in pediatric supplementation. Second, FontUp® has demonstrated superior bioavailability, exhibiting a longer half-life, enhanced gastrointestinal stability, and reduced inter-individual variability in plasma concentrations compared to EGCG alone (Fernández et al., 2020). Although the Font-Up® formulation contains additional components such as vitamins and minerals, their concentrations are more than an order of magnitude below those required to significantly affect neurocognitive or physiological outcomes (Mengelberg et al., 2022; Sim et al., 2022; Zhou et al., 2023).

### Intervention

FASD participants received an oral dose of 9 mg/kg/day of EGCG via FontUp®, a cocoa-flavored nutritional powder dissolved in 200 mL of semi-skimmed milk. The 9mg/kg/day dose is grounded in prior pediatric research (Cieuta-Walti et al., 2022; de la Torre et al., 2016), and a maximum daily limit of 400 mg. The product was purchased from Grand Fontaine Laboratories (Barcelona, Spain).

### FASD diagnosis

The diagnosis and clinical assessment were made by a pediatrician and a clinical psychologist specialized in FASD, following the Updated Clinical Guidelines for Diagnosing FASD by Hoyme et al. (2016). Briefly, each child was assigned to a FASD diagnostic category according to the classification of the 1996 IOM criteria (reviewed in 2016) (Hoyme et al., 2005, 2016), based on 5 diagnostic characteristics: 1) confirmed prenatal alcohol exposure; 2) evidence of a characteristic minor facial abnormalities pattern (2), typified by having a thin upper lip, smooth philtrum and short palpebral fissures; 3) growth retardation, defined as height or weight (1) ≤10th percentile; 4) evidence of deficient brain growth or subrogated data; and 5) behavioral or cognitive affected domains (1 or 2) related to prenatal alcohol exposure. For a diagnosis of complete FAS, at least criteria 2, 3, 4, 5 (confirmed or not confirmed PAE) were required. For partial FAS, criteria 1, 2, and at least one of criteria 5 (confirmed PAE) or 2, 5, and 3 or 4 (no confirmed PAE) were required. The diagnosis of alcohol-related birth defects (ARBD) required the finding of 1 criterion plus a minimum of one structural defect involving heart, skeleton, kidney, eye, ear, or minor abnormalities like railway ears, midface hypoplasia, or stick hockey hands. The diagnosis of alcohol-related neurodevelopmental disorders (ARND) required the finding of 1 and 5 criteria.

### Blood collection, processing and storage protocol

A total of 5 mL of whole blood was collected from both control and FASD group at baseline using BD Vacutainer® SST® II Advance® (BD Biosciences; 367,955). For the FASD group, additional blood samples were obtained 12 months post-treatment. Immediately after collection, the blood samples were centrifuged at 1750 g for 10 min. Serum samples were stored at −80 °C until further analysis.

A further 5 mL of blood was collected into BD Vacutainer® K3E 15% Aprotinin 250KIU tubes (SGSH; BD361017). Peripheral blood mononuclear cells (PBMCs) were isolated from whole blood by centrifugation using Blood Collection tube BD Vacutainer®, BD CPT™ (Avantor, BDAM362781) at 1750 g for 25 min. After isolation, PBMCs were washed twice with PBS at 500 g. RNA was extracted from the PBMCs using the QIAshredder (Qiagen, 79,654) and RNAsy Mini-Kit (Qiagen, 74,104) protocols for RNA isolation. The RNA was quantified using Nanodrop One (Thermo Fisher Scientific). Nucleic acids included in the study had a 260/280 ratio and a 260/260 ratio >1.8. RNA samples were stored at −80 °C for further analysis.

### Luminex / multiplex bead-based immunoassay

Serum samples were centrifuged at 16,000 g for 4 min and then diluted 1:2 and 1:4 with Calibrator Diluent RD6–40 following the manufacturer’s instructions. The concentration of several biomarkers, IL-6, CCL2, CX3CL1, IL-10, NGFβ, IL1β, IFNγ, CXCL16, VCAM-1, MMP-10, were quantified in serum samples using a personalized human multiplexed magnetic beads-based immunoassay (LXSAHM-06 and LXSAHM-08, R&D Systems). Serum samples (50 μL/well) or standards (50 μL/well) were incubated according to the manufacturer's instructions.

The standard for each biomarker was provided by the manufacturer and was used to establish standard curves to maximize the sensitivity of the assay. Biomarker levels were determined using a Luminex analyzer (Luminex 200) and the data were reported accordingly. More detailed information can be found in the original protocol provided by R&D Systems.

### Reverse transcription-quantitative real-time PCR (RT-qPCR)

Reverse transcription was performed using the Applied BiosystemsTM High Capacity cDNA Reverse Transcription Kit (Fisher Scientific; 10,400,745) according to the manufacturer's protocol. RT-qPCR was performed in 384-well plates (Axygen, PCR-384M2-C) using PerfeCTa SYBR Green FastMix (Quantabio, 95,074–012) in QuantStudioTM 7 Real-Time PCR (Thermo Fisher Scientific; 4485,701). The GAPDH gene was used for normalization. Primers for the analyzed genes (*CCL2, CX3CL1, CXCL16, IL-1β, IL-10, IL-6, VCAM-1, MMP-10, IFNγ, enolase-2, NGFβ* and *S100β*) were provided by Sigma Aldrich and are listed in Supplementary Table 2. The RNA used was obtained from the same samples subjected to RNA-seq. The 2−ΔΔCt method was used to assess relative abundance.

### Statistical analysis

Results were expressed as mean and standard deviation. Comparisons were performed between controls and FASD as well as between FASD and FASD after EGCG treatment. Data were first tested for normality using the Shapiro-Wilk test. One-way ANOVA followed by Tukey's multiple comparison test was used to assess differences between groups. For non-normally distributed samples, Kruskal-Wallis test was used, followed by Dunn's multiple comparison test.

In addition to the aforementioned statistical tests, ML algorithms were also employed to build a predictive model. Significant biomarkers included in this study were used as variables for the model: CCL2, CX3CL1, CXCL16, IL-1β, IL-10, IFNγ, and NGFβ.

All statistical analyses were performed using the R statistical package. All graphs were generated using GraphPad Prism software.

### Machine learning models

This research tested a variety of ML algorithms, including Logistic Regression (LR), Linear Discriminant Analysis (LDA), Support Vector Machine (SVM) in both its linear and polynomial forms, Decision Tree (DT), K-Nearest Neighbors (KNN), Random Forests (RF), and Extreme Gradient Boosting (XGB), to predict FASD.

The analysis incorporated significant biomarkers evaluated in this study (7), namely CCL2, CX3CL1, CXCL16, IL-10, IFNγ, and NGF. Each biomarker was tested for outliers using Rosner’s generalized extreme Studentized deviate test, implemented through the rosnerTest function from the EnvStats package. The dataset was subsequently scaled using ‘scale’ function in base R. Prior to model construction, a hold-out method was applied to split the data into training and test sets using ‘createDataPartition’ function from *caret* package in R (Kuhn, 2008). 67% of the data was allocated to training set and the remaining 33% to test set. This function employs a stratified random sampling method, which minimizes the bias of the data distribution and creates balanced data.

In addition to the hold-out method, a resampling method involving 5-fold cross-validation and three repeats was adopted. This was implemented using ‘trainControl’ function from the *caret* package (Kuhn, 2008). The models were trained using ‘train’ function with hyperparameters set to default, which gathers and simplifies numerous R algorithms for the development of predictive models (Kuhn, 2008). The models employed included LR, using ‘glm’ method and binomial family, and LDA, implemented with ‘lda’ method, which has ‘moment’ as the default mean and variance estimator. Linear SVM and Polynomial SVM were performed using ‘svmLinear’ and ‘svmPoly’ methods, respectively. They have C tuning parameter, which determines the margin classification, equal to 1 as default settings. KNN was employed by ‘knn’ method also from *caret* package, performing automatic hyperparameter tuning for k depending on instance-based learning. In addition, RF was employed using ‘rf’ method, with 500 trees as default. XGB model used ‘xgbTree’ method, having 100 maximum iterations by default.

The ‘predict’ function from *stats* package was used to predict classes with the test group. In order to make comparisons, the ‘confusionMatrix’ function from *caret* package was used to calculate true positive, true negative, false positive, and false negative. These calculations provided measures including accuracy, precision, sensitivity, F1 score and specificity. ROC-AUC was obtained using ‘roc’ function from *pROC* package (Robin et al., 2011). Training and test datasets were consistent across FASD and its subgroups, ensuring a fair and valid comparison.

Feature importance prediction of the models was determined by calculating the Root Mean Square Error (RMSE) loss after permutation. It was obtained with ‘explain’ function from *DALEX* package, with ‘classification’ type model in arguments (Law Biecek, 2018). Plots were generated from the object class formed by ‘variable_importance’ function from *caret* package (Kuhn, 2008).

LR is a binary classification method that uses a logistic function for class probability estimation and maximum likelihood estimation for coefficient assessment. LR is easy to implement, but can overfit with many features. LDA reduces data dimensionality, maximizing class separability, to identify a linear combination of features that characterizes a group. SVM maximizes the distances between class-separating hyperplanes. We differentiate between linear SVM (efficient, for linearly separable data) and polynomial SVM (for non-linear data, computationally intense). DT is a non-parametric, tree-structured algorithm that splits data into subsets based on the value of input features, and handles both numerical and categorical data but is prone to overfitting if not properly pruned. KNN predicts class by calculating the Euclidean distance to training points, selecting K most similar instances. Its performance degrades on high-dimensional datasets. Ensemble methods such as RF and XGB are based on decision trees. RF combines multiple independently trained decision trees, uses bagging to create subsets of the original dataset, and then aggregates the results. XGB, on the other hand, trains decision trees sequentially, with each new tree correcting the errors made by the previous one. Our study aimed to identify the most effective model for predicting FASD by comparing different models. This variety of approaches enhanced the robustness and comprehensiveness of the study.

To explore the multidimensional structure of our data, we applied t-distributed Stochastic Neighbor Embedding (t-SNE) using the Rtsne package in R, setting three dimensions and a perplexity value of 20 to balance local and global structure preservation. To analyze group separability, we calculated centroids for each group by computing the mean t-SNE coordinates across all three dimensions. A Multivariate Analysis of Variance (MANOVA) was performed to determine whether the group centroids differed significantly. Pairwise comparisons were conducted using Bonferroni-adjusted *t*-tests to identify specific group differences. T-SNE visualization was performed using plotly package.

## Results

**Fig. 1:**
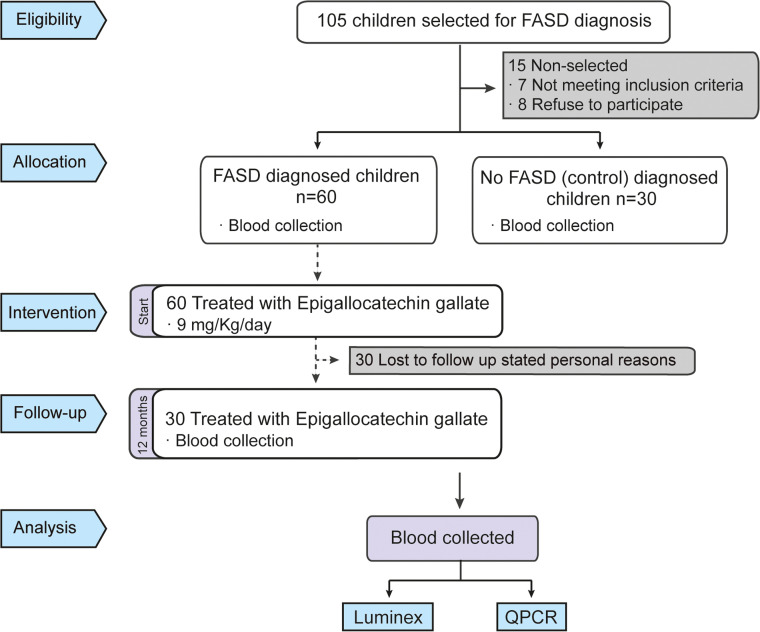
**Flowchart diagram**. Flowchart of FASD diagnosis and EGCG study in FASD patients during 12 months.

A total of 105 subjects from different previous cohorts of EEC adoptees with a suspicion of FASD diagnosis were initially enrolled in the study. Among these, 7 subjects did not meet the inclusion criteria, and 8 declined participation, leaving 90 subjects who underwent neurocognitive and clinical evaluation for FASD diagnosis (Fig. 1).

Out of the 90 evaluated, 60 were diagnosed with FASD, while the remaining 30 were categorized as unaffected controls. None of the control participants had a documented history of prenatal alcohol exposure, and their neuropsychological assessments and clinical evaluations did not indicate any impairment.

30 children diagnosed with FASD completed the 12-month EGCG treatment regimen (Fig. 1). Blood samples were collected at baseline from both the FASD and control (No FASD) groups, and again after 12 months of treatment for the FASD group. 30 patients were lost to follow-up due to a combination of personal, behavioral, psychosocial, and procedural challenges commonly encountered in individuals diagnosed with FASD and their families. Comparative analysis of the FASD and control groups showed no significant differences in age, sex, or the prevalence of pre-existing comorbidities, as summarized in Table 1.

**Table 1:** Distribution by sex and mean age of participants.

	**Control (*n*=30)**	**FASD**	
		**FAS (*n*=47)**	**pFAS (*n*=13)**
**Descriptive characteristics**			
Sex (n, % male)	63 (19)	45 (21)	62 (8)
Age (years)	12.9 (3.7)	11.3 (4.0)	14.6 (4.0)
**Anthropometric measurements**			
Height (cm)	150.3 (15.9)	142.5 (17.0)	158.2 (14.5)
Weight (kg)	43.8 (12.1)	39.6 (12.1)	50.9 (11.9)
BMI (kg/m2)	28.6 (4.8)	27.2 (5.1)	31.8(4.7)

Data for age, height, weight, and BMI are represented by mean and SD.

Abbreviations: BMI, Body Mass Index; FAS, Fetal Alcohol Syndrome; SD, Standard Deviation; pFAS, partial Fetal Alcohol Syndrome.

### Serum biomarker analysis and EGCG effect in FASD

**Fig. 2:**
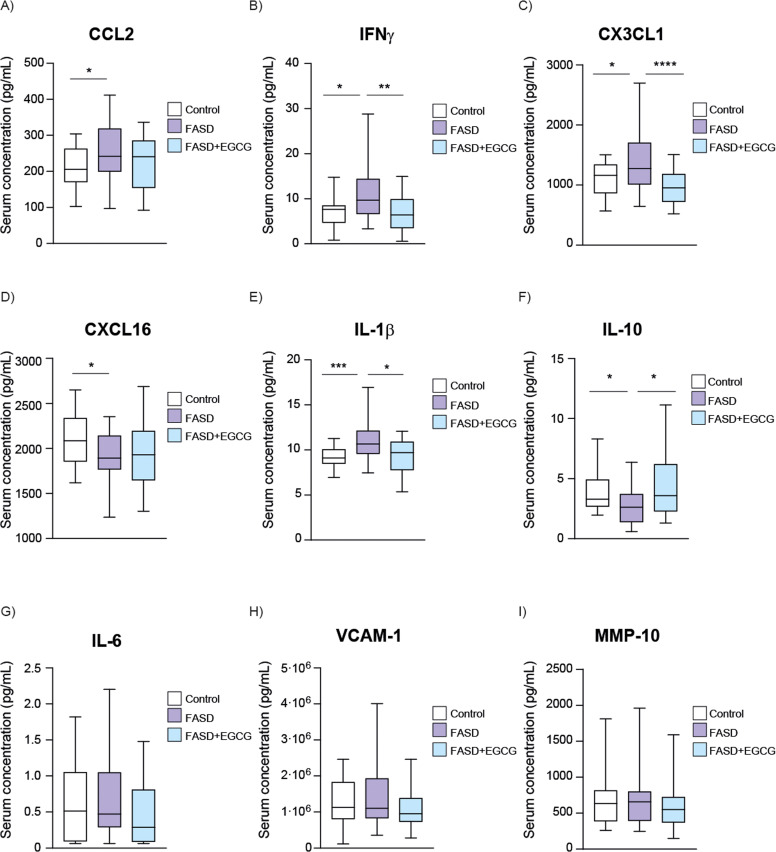
**Boxplot of serum concentration (pg/mL) of inflammatory and immune response biomarkers.** (A) IFNγ, (B) CX3CL1, (C) IL-1β, (D) CCL2, (E) IL-10, (F) CXCL16, (G) IL-6, (H) VCAM-1, (I) MMP-10. White represents control patients; purple represents FASD patients and blue represents FASD patients at 12 months of EGCG treatment. **** *P* < 0.001, *** *P* < 0.005, ** *P* < 0.01, * *P* < 0.05.

Serum biomarker concentrations were quantified using Luminex multiplex immunoassays showing a significant increase in the level of IFNγ, CX3CL1, IL-1β and CCL2 in FASD group compared to controls (Fig. 2A–D, Supplementary Table 3). After EGCG treatment, IFNγ, CX3CL1, and IL-1β levels were significantly recovered to physiological levels in FASD patients (Fig. 2A–C). Although a reduction in CCL2 levels was observed after EGCG treatment, this change did not reach statistical significance (Fig. 2D). In contrast, IL-10 and CXCL16 levels were significantly lower in the FASD group (Fig. 2E, F) compared to control group. EGCG treatment successfully restored IL-10 levels. However, no significant differences were detected in CXCL16 levels.

IL-6, VCAM-1, and MMP10 levels trended upward in the FASD group compared to controls (Fig. 2G–I), though not statistically significant. Although EGCG treatment resulted in a slight decrease in these biomarkers, the changes were not statistically significant.

**Fig. 3:**
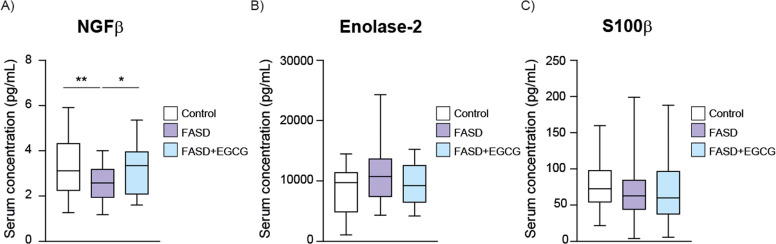
**Boxplot of serum concentration (pg/mL) of neuronal biomarkers**. (A) NGFβ, (B) Enolase-2, (C) S100β. White represents control patients; purple represents FASD patients and blue represents FASD patients at 12 months of EGCG treatment. **** *P* < 0.001, *** *P* < 0.005, ** *P* < 0.01, * *P* < 0.05.

Regarding neuronal biomarkers, a significant decrease in NGFβ was observed in the FASD group (Fig. 3A), suggesting that PAE may downregulate NGFβ expression, potentially contributing to the neurodevelopmental impairments associated with FASD. The FASD group treated during 12 months with EGCG exhibited a significant increase in NGFβ levels compared to the FASD group, then restoring the physiological levels. Moreover, no significant differences were observed between groups for Enolase-2 and s100β biomarkers (Fig. 3B, C).

**Fig. 4:**
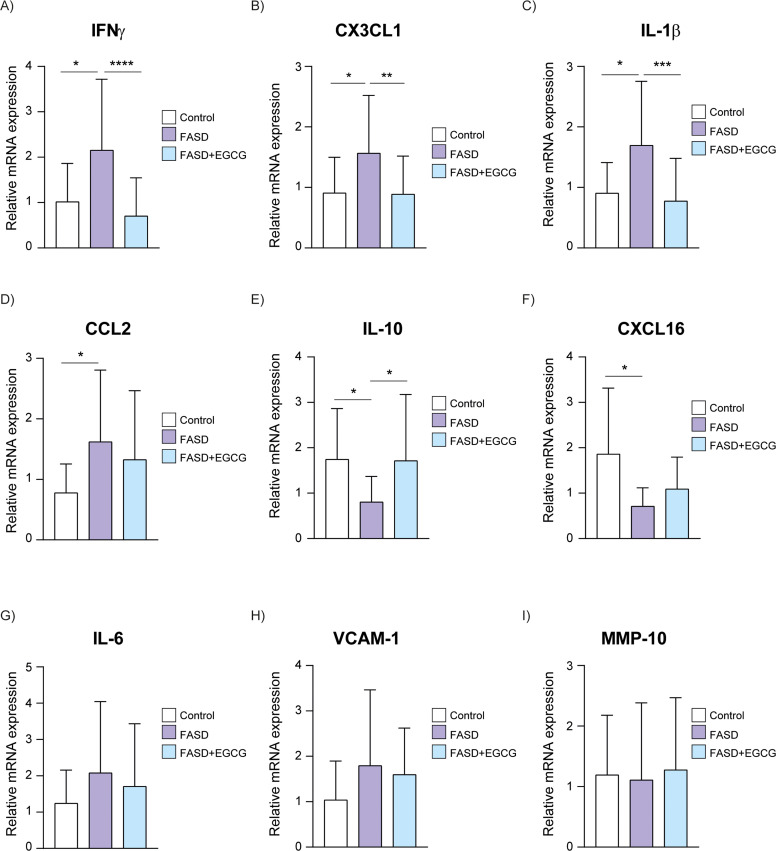
**RT-qPCR of selected inflammatory and immune biomarkers.** Validation of inflammatory and immune biomarkers in control, FASD and FASD+EGCG groups. (A) IFNγ, (B) CX3CL1, (C) IL-1β, (D) CCL2, (E) IL-10, (F) CXCL16, (G) IL-6, (H) VCAM-1, (I) MMP-10. White represents control patients; purple represents FASD patients and blue represents FASD patients at 12 months of EGCG treatment. **** *P* < 0.001, *** *P* < 0.005, ** *P* < 0.01, * *P* < 0.05*.

To validate these findings, gene expression of several biomarkers was analyzed using RT-qPCR. there was a significant increase in IFNγ expression in the FASD group compared to the control, with a subsequent significant reduction observed following EGCG treatment (Fig. 4A). Similarly, CX3CL1 mRNA expression were significantly elevated in the FASD group compared to controls and EGCG significantly reduced CX3CL1 expression in the FASD+EGCG group (Fig. 4B). IL-1β expression showed significantly increased expression in the FASD group compared to the control and significantly reduced expression after EGCG treatment (Fig. 4C). The mRNA expression of CCL2 exhibited trends consistent with the Luminex assay results. A significant increase was observed in the FASD group compared to controls (Fig. 4D), followed by a decreasing trend after EGCG treatment, although it was not significant. IL-10 expression was also reduced in FASD group and significantly increased after EGCG treatment, suggesting an anti-inflammatory effect (Fig. 4E). Similarly, CXCL16 mRNA expression was reduced in FASD group compared to controls (Fig. 4F). An increasing trend in CXCL16 expression was observed after EGCG treatment, although it was not statistically significant. According to immunoassays results, IL-6, VCAM-1 and MMP-10 expression did not show significant differences among the groups (Fig. 4G–I).

**Fig. 5:**
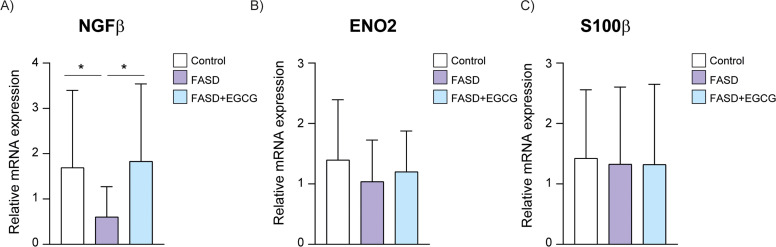
**RT-qPCR of selected neuronal biomarkers.** Validation of neuronal biomarkers in Control, FASD and FASD+EGCG. (A) NGFβ, (B) Enolase-2, (C) S100β. White represents control patients; purple represents FASD patients and blue represents FASD patients at 12 months of EGCG treatment. * *P* < 0.05.

Regarding neuronal biomarkers, NGFβ mRNA expression exhibited a significant decrease in the FASD group compared to controls (Fig. 5A). EGCG treatment resulted in a significant increase of NGFβ expression compared to FASD group. Consistent with the Luminex results, no significant differences were detected among the groups for Enolase-2 and s100β mRNA expression (Fig. 5B, C).

Therefore, the RT-qPCR analysis corroborated the findings obtained from the Luminex immunoassays, highlighting the potential of EGCG to mitigate the neuroinflammatory and neurodevelopmental disruptions through the normalization of specific biomarkers.

### Predictive model

Predictive models were developed using 7 biomarkers, which include CCL2, IFNγ, CX3CL1, CXCL16, IL-1β, IL-10, and NGFβ, selected based on their statistically significant group differences and established biological relevance in the context of FASD-related neuroinflammation and immune dysregulation. This targeted feature selection strategy was applied to reduce dimensionality, minimize the risk of overfitting in a limited dataset, and enhance the interpretability and clinical applicability of the resulting models.

**Fig. 6:**
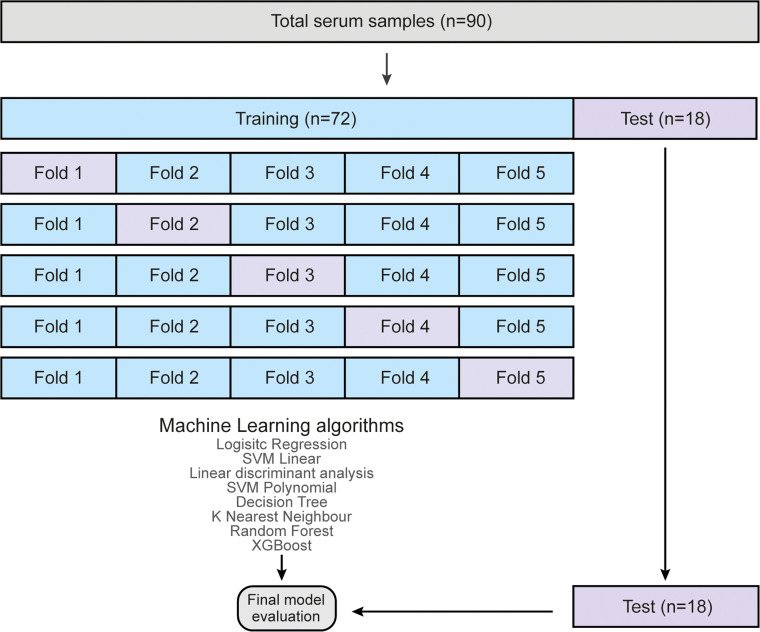
**Predictive model flowchart**. Machine learning model development structure for FASD prediction.

A total of 90 samples were included in the study, with 72 samples allocated for training the models and 18 samples reserved for testing and final model evaluation (Fig. 6). Multiple ML algorithms were performed, including LR, SVML, LDA, SVMP, DT, KNN, XGB, and RF. All models were trained using 5-fold cross-validation on the training dataset, and the final model performance metrics on the test dataset are summarized in Table 2.

**Table 2:** Hyperparameters and test performance.

**Performance Measures**	**Machine Learning Models**							
	**LR**	**SVM Linear**	**LDA**	**SVM Polynomial**	**Decision Tree**	**KNN**	**XGBoost**	**Random Forest**
**Accuracy**	0.72	0.78	0.78	0.78	0.61	0.61	0.78	0.89
**Precision**	0.77	0.83	0.83	0.83	0.86	0.67	0.83	0.92
**Sensitivity**	0.83	0.83	0.83	0.83	0.5	0.83	0.83	0.92
**F1 Score**	0.8	0.83	0.83	0.83	0.63	0.74	0.83	0.92
**Specificity**	0.5	0.67	0.67	0.67	0.83	0.17	0.67	0.83
**ROC AUC**	0.67	0.75	0.75	0.75	0.67	0.5	0.75	0.88

Abbreviations: LR, Logistic Regression; SVML, Support Vector Machine Linear Kernel; LDA, Linear Discriminant Analysis; SVMP, Support Vector Machine Polynomial Kernel; KNN, k-Nearest Neighbour; XGB, gradient-boosted trees; RF, Random Forest; AUC, Area Under the Curve.

**Fig. 7:**
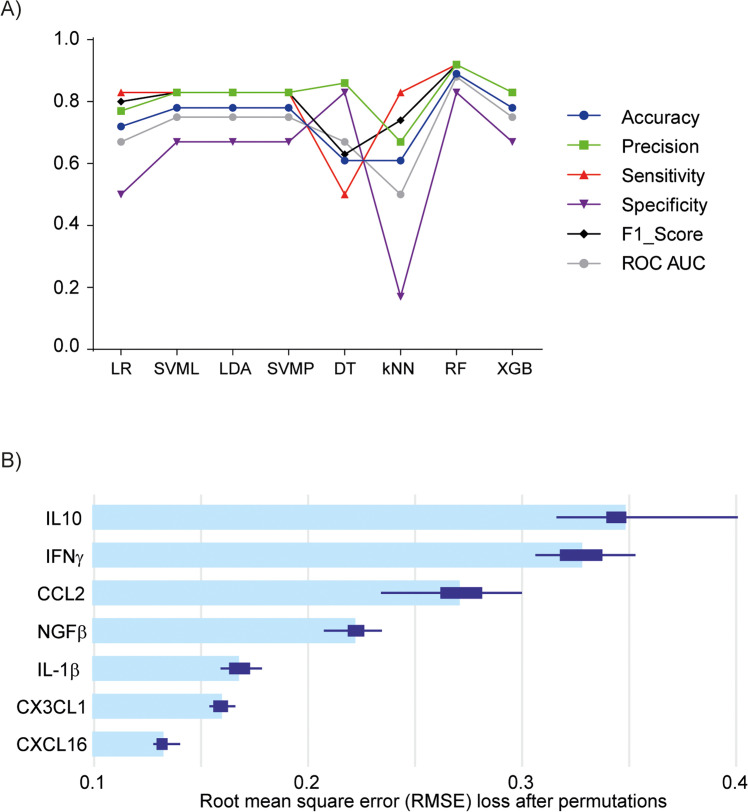
**Machine learning algorithm modeling.** (A) Model performance for FASD prediction. (B) Mean variable-importance of RF model for FASD prediction, calculated using 50 permutations and the root-mean-squared-error-loss-function. LR, Logistic Regression; SVML, Support Vector Machine Linear Kernel; LDA, Linear Discriminant Analysis; SVMP, Support Vector Machine Polynomial Kernel; DT, Decision Tree; KNN, k-Nearest Neighbor; XGB, gradient-boosted trees; RF, Random Forest.

Among the models, RF demonstrated the highest predictive performance, achieving the highest accuracy (0.89), precision (0.92), sensitivity (0.92), F1 Score (0.92), specificity (0.83), and ROC AUC (0.88), making it the most effective model for predicting FASD diagnosis. Other models such as Logistic Regression, SVML, LDA, SVMP, Decision Tree, KNN, and Xgboost showed lower performance across these metrics (Table 3, Fig. 7A). Based on these results, the RF model was selected as the primary predictive tool for FASD diagnosis due to its robust discriminative ability and superior overall performance.

**Table 3:** **Centroid coordinates of control, FASD, and EGCG groups in the t-SNE space.** The table presents the mean centroid positions (X, Y, and Z coordinates) for each group based on the t-SNE dimensionality reduction.

Group	Control	FASD	FASD+EGCG
X centroid	0.6268624	−1.2104418	1.7940211
Y centroid	−2.896919	2.667445	−2.437972
Z centroid	−1.4199625	−0.0198971	1.45975662

To understand the decision-making process of RF model, we analyzed the importance of the variables in this algorithm. Feature importance was determined by calculating the Root Mean Square Error (RMSE) loss after permutation. The features were ranked in order of importance, with IL-10 (0.35) having the highest importance, IFNγ (0.33), CCL2 (0.27), NGFβ (0.22), IL-1β (0.17), CX3CL1 (0.16), and CXCL16 (0.12) (Fig. 7B). These results provide valuable insights into the key serum biomarkers involved in FASD conditions and their relative importance in the predictive model.

Partial dependence plots were generated to illustrate the influence of individual biomarkers on the predictions made by RF. These plots provide a visualization of the marginal effect of each biomarker on the predicted probability of FASD diagnosis, averaged across all other features in the dataset. The x-axis represents the range of scaled biomarker levels, while the y-axis indicated the marginal predicted probability for FASD. An upward trend in the plot suggests that higher levels of the biomarker increase the likelihood of an FASD diagnosis, whereas a downward trend suggests that lower biomarker levels are associated with a higher probability of diagnosis.

**Fig. 8:**
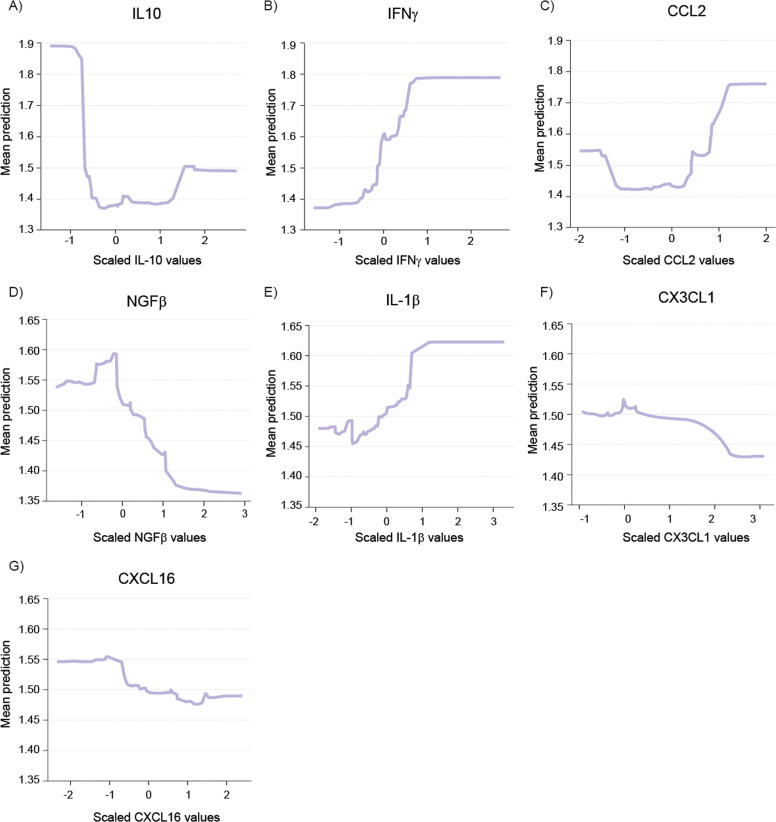
**Partial dependence plot (PDP) from RF model.** PDP illustrates the influence of biomarker levels in model predictions. A downward trend indicates that higher values of the biomarker decrease the likelihood of being classified as FASD, whereas an upward trend suggests a higher likelihood of FASD classification with increasing biomarker levels. CCL2, C—C Motif Chemokine Ligand 2; CX3CL1, C-X3-C Motif Chemokine Ligand 1; CXCL16, C-X-C Motif Chemokine Ligand 16; IL-1β, Interleukin 1 Beta; IL-10, Interleukin 10; IFNγ, Interferon Gamma; NGFβ, Nerve Growth Factor Beta. X-axis represents the scaled values of each biomarker. Y-axis represents the mean predicted value for the FASD risk.

Aligning with Luminex results, the analysis of IL-10 revealed that a decrease in its levels was associated with a marked increase in the predicted probability of FASD diagnosis (Fig. 8A). For IFNγ and CCL2, higher levels of this biomarker were associated with increased predictions for FASD (Fig. 8B, C). NGFβ levels demonstrated a negative relationship with FASD predictions, with lower levels indicating a higher probability of diagnosis (Fig. 8D). IL-1β showed a strong positive association with FASD predictions (Fig. 8E). However, CX3CL1 and CXCL16 showed a less pronounced effect (Fig. 8F, G), being the two less important variables for FASD prediction.

**Fig. 9:**
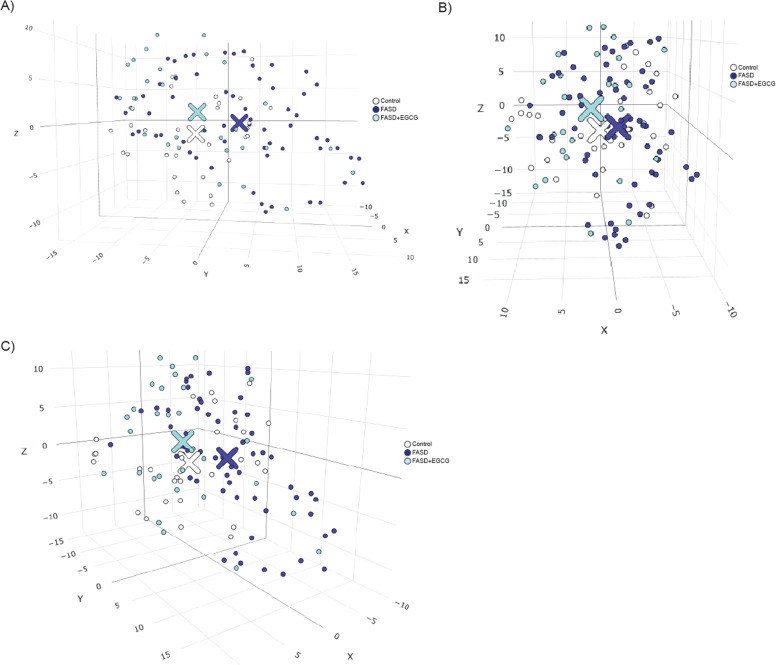
**Three-dimensional t-SNE clustering analysis of control, FASD, and FASD+EGCG samples.** The T-distributed Stochastic Neighbor Embedding (t-SNE) visualization illustrates the similarity relationships among the samples. Control is represented in white, FASD in purple, and FASD+EGCG in blue. The spatial distribution of the clusters highlights the separation between groups, with the EGCG-treated samples appearing to shift towards the control cluster, suggesting a potential effect of the treatment. Figures A, B and C visualize different positions of the t-SNE.

To further examine how EGCG treatment modulates biomarker profiles, a dimensionality reduction approach using t-distributed Stochastic Neighbor Embedding (t-SNE) was employed to visualize the clustering patterns of control, FASD, and EGCG-treated FASD patients (Fig. 9, Additional HTML File). To determine whether EGCG treatment shifts FASD patients toward a control-like profile, the centroids (mean positions) of each group in the t-SNE reduced space were compared (Table 3). A Multivariate Analysis of Variance (MANOVA) was performed to assess overall differences among centroids across groups, revealing a statistically significant difference (*p* < 0.001), indicating that at least one group centroid is significantly different from the others.

To further dissect these differences, pairwise *t*-tests were conducted for each of the three t-SNE dimensions (X, Y, Z), with Bonferroni correction applied for multiple comparisons. The results revealed significant centroid shifts in the X dimension between FASD and FASD+EGCG (*p* = 0.03) (Supplementary Table 4), suggesting that EGCG-treated patients exhibit a biologically relevant movement away from untreated FASD individuals. Significant differences in the Y dimension between FASD and FASD+EGCG (*p* = 0.016) and between FASD and control (*p* = 0.007) were observed, with no significant difference between control and FASD+EGCG (*p* = 1.000) (Supplementary Table 5). This suggests that EGCG treatment aligns the Y-component biomarker profile of FASD patients more closely with control individuals. No statistically significant differences in the Z dimension, implying that EGCG did not fully normalize all aspects of the biomarker profile (Supplementary Table 6).

The 30 FASD patients treated with EGCG were classified using the RF model previously discussed; 11 were classified as control and 19 as FASD, suggesting that a subset of EGCG-treated individuals exhibit a biomarker profile that is more similar to control than untreated FASD, confirming the recovery of physiological levels. The probabilities can be found in Supplementary Table 7. However, the persistence of FASD-like classification in some EGCG-treated individuals suggests that additional factors influence biomarker normalization.

## Discussion

PAE disrupts critical physiological pathways, including neuroinflammation, oxidative stress, and hormonal imbalances, leading to lifelong cognitive, behavioral, and physical impairments (Kane & Drew, 2021; Popova et al., 2023). Despite advances in understanding the pathophysiology of FASD, traditional diagnostic methods remain limited by their reliance on clinical features and confirmation of maternal alcohol, which further complicates accurate diagnosis. These challenges highlight the need for innovative diagnostic strategies that take into account the heterogeneity of FASD manifestations. Over the past decade, research on early diagnostic biomarkers for neurodegenerative diseases, including Alzheimer's and Parkinson', has highlighted the potential of serum-based biomarkers due to their cost-effectiveness, time-efficiency, and non-invasiveness, compared to cerebrospinal fluid (Ausó et al., 2020; Koníčková et al., 2022; Le et al., 2017). Technologies like the Luminex assay allow the simultaneous measurement of multiple biomarkers, providing insights into molecular disruptions caused by PAE. Previous studies revealed impaired levels of fractalkine (CX3CR1) (Roberson et al., 2011) and mTOR signaling proteins and phosphoproteins (de la Monte et al., 2023). Another study showed that Insulin-like Growth Factor-I (IGF-I) and Insulin-like Growth Factor-II (IGF-II) levels were significantly lower in FASD and prenatal ethanol exposure (PEE) compared to the control group (Andreu-Fernández et al., 2019). Furthermore, research on effective treatments for FASD is essential to alleviate symptoms and improve the quality of life of individuals affected. However, the potential for novel therapeutic interventions remains largely unexplored. One promising treatment is the use of the natural antioxidant EGCG, which has demonstrated anti-inflammatory, antioxidant, and cognitive stimulation properties (de la Torre et al., 2016). The present study investigated not only the biomarker profile of critical pathways associated with FASD, but also the effect of EGCG treatment on these biomarkers related to FASD physiopathology.

Some studies have highlighted the association between cytokine and chemokine alterations and FASD in both neonatal and adult brains of FASD-mouse models, producing immune dysregulation and inflammation (Bodnar et al., 2016, 2020; Noor & Milligan, 2018). Consistent with these findings, this study identified elevated levels of CCL2, IFNγ, and CX3CL1 in patients diagnosed with FASD. These molecules play critical roles in inflammatory processes, neuronal and microglial dysfunction, and impaired neurogenesis, as confirmed by previous research (Bachstetter et al., 2011; Bodnar et al., 2016; Chang et al., 2018; Gao & Ji, 2010; Ginhoux et al., 2010; Pascual et al., 2017; Sanford & McEwan, 2022; Zhang & Luo, 2019). Notably, CCL2, IFNγ, and CX3CL1 are known to mediate the recruitment and activation of monocytes and microglia, contributing to the release of neurotoxic factors for the CNS (Deshmane et al., 2009; Tau & Rothman, 1999). Importantly, EGCG treatment reduced the levels of these inflammatory markers, indicating its potential to modulate the inflammatory cascade and provide neuroprotection by suppressing key regulators such as Nuclear Factor Kappa B (NF-kB) and Tumor Necrosis Factor alpha (TNF-α) (Bosch-Mola et al., 2017; Lee et al., 2009; Li et al., 2012; Melgarejo et al., 2009; Wang et al., 2012).

Conversely, CXCL16 chemokine showed reduced levels in patients diagnosed with FASD. CXCL16 is a chemokine that regulates innate immunity and provides neuroprotection against damage, inflammation, and hypoxia (Lepore et al., 2018; Rosito et al., 2012, 2014). The observed reduction in CXCL16 levels likely reflects the neurotoxic impact of PAE, consistent with findings in animal models of PAE (Juárez-Rodríguez et al., 2020). Our results showed that treatment with EGCG did not significantly restore CXCL16 levels, suggesting that its therapeutic effects are more likely attributable to the suppression of pro-inflammatory mediators rather than the re-establishment of CXCL16 chemokine homeostasis. This finding aligns with previous studies reporting that EGCG had no effect on CXCL16 cytokine levels (Li et al., 2011). However, other investigations have observed a reduction in CXCL16 levels following EGCG treatment, both *in vivo* (Saleh et al., 2015) and *in vitro* (Saleh et al., 2013), suggesting that its impact on CXCL16 may be context-dependent.

PAE alters the hypothalamic-pituitary-adrenal (HPA) axis, a critical regulator of the immune and neuroendocrine systems, with interleukins playing a pivotal role in this dysregulation (Bodnar et al., 2016, 2020; Ruffaner-Hanson et al., 2023). Among these, IL-1β plays a proinflammatory role in acute and chronic inflammatory disorders (Ren & Torres, 2009). Our findings indicated that patients diagnosed with FASD had higher levels of IL-1β compared to the controls, which is consistent with previous research suggesting that PAE conditions activate microglia and release pro-inflammatory molecules, such as IL-1β (Lawrimore et al., 2019; Mukherjee et al., 2023; Pascual et al., 2017). Our results also showed a significantly reduced expression of IL-10, an anti-inflammatory interleukin (Moore et al., 2003). IL-10 modulates immune responses by limiting excessive inflammation and preventing tissue damage (Iyer & Cheng, 2012). It is primarily produced by regulatory T cells, monocytes, macrophages, and dendritic cells in response to inflammatory stimuli (Iyer & Cheng, 2012). Our results are consistent with studies examining children with a history of PAE at 6 and 12 months of age (Bodnar et al., 2020), and FASD-rat model observations (Noor et al., 2016). Reduced IL-10 levels may lead to an exaggerated pro-inflammatory response, increasing susceptibility to neuroinflammation and immune dysregulation, potentially influencing developmental outcomes (Noor et al., 2016). Furthermore, studies revealed that PAE potentiated proinflammatory interleukins such as IL-1β and simultaneous suppression of IL-10 (Noor et al., 2016). EGCG therapy significantly restored IL-1β and IL-10 levels, by reducing the phosphorylation of the NF-kB p65 subunit (Sah et al., 2022), demonstrating its anti-inflammatory potential as previously reported (Shan et al., 2018; Wheeler et al., 2004). Interestingly, a study observed the downregulation of IL-1β in embryos after treatment with the antioxidant astaxanthin in FASD mice (Zheng et al., 2014).

Although previous studies have reported elevated levels of IL-6, a cytokine with both anti- and pro-inflammatory properties, after alcohol exposure (Noor & Milligan, 2018; Roberson et al., 2012; Schnur et al., 2023), our study did not show statistically significant differences in these levels. However, our findings demonstrated a broader trend of upregulation of other interleukins in FASD patients, indicative of heightened inflammatory responses and disruptions in HPA axis regulation. The increased production of pro-inflammatory molecules in FASD leads to increased trans-endothelial leukocyte migration across the blood spinal-cord barrier, producing damage in CNS (Noor & Milligan, 2018). VCAM-1, a cell adhesion protein primarily expressed in endothelial cells, plays a pivotal role in these inflammatory processes (Kong et al., 2018). Previous studies reported that PAE upregulated adhesion molecules such as VCAM-1, facilitating leukocyte migration into the CNS and exacerbating neuroinflammation [26,29]. However, no significant changes in VCAM-1 levels were observed in our study. Similarly, vascular remodeling proteins like MMP-10, which have been reported to be reduced in FASD and may confer immune-protective properties (Gano et al., 2020; Rosenberg et al., 2010), did not show significant differences in our cohort.

Neurotrophins, which are essential for brain development and synaptic plasticity, play a crucial role in maintaining neuronal function and survival (Carito et al., 2019). NGFβ, a neurotrophic factor, is expressed both in CNS and peripheral nervous system, where it regulates neuronal maturation and survival (Bersani et al., 2000). Abnormalities in NGFβ levels during fetal growth can have long-lasting effects on neuroplasticity, learning, memory, and behavior (Carito et al., 2019). Our study found a significant decrease in NGFβ in FASD group. Previous research suggests that alcohol exposure leads to promoter methylation of NGFβ gene, leading to a significant decrease in NGFβ expression, producing the neurodevelopmental impairments observed in FASD (Heberlein et al., 2013). In contrast, biomarkers levels of enolase-2, a marker of neuronal maturation whose levels were reduced in PAE rat model (Ledig et al., 1990), and s100β, a potential biomarker for brain or spinal cord injury (Yokobori et al., 2013), were not significantly different in our study. Interestingly, after EGCG treatment, NGFβ levels increased significantly in the FASD group, reaching levels comparable to the control group. This finding suggests a potential neurostimulatory effect of EGCG, probably due to stimulation of neuritogenic activity, as observed in previous studies (Gundimeda et al., 2010). These results highlight the specificity of EGCG's effects on certain neurotrophic factors that could provide an answer to the improvement of cognitive function, such as memory and learning tasks, observed in FASD-mouse model (Tiwari et al., 2010). However, our results also suggest a limited effect on other biomarkers associated with neuronal growth and astrocytic function.

In order to evaluate the effects of EGCG on biomarker expression, a multivariate analysis of t-SNE centroids was performed. Significant differences were observed between the untreated FASD group and the FASD+EGCG group along the Y dimension, while no significant differences were detected between the EGCG-treated group and the control group across the X, Y, and Z dimensions. These findings suggest that EGCG treatment shifts the biomarker profile of FASD patients toward a closer control-like state. Consistent with this, our ML model identified 58% of the FASD+EGCG group as belonging to the control group. This result highlights the efficacy of EGCG in modulating key molecular pathways affected by PAE, reinforcing its potential as a therapeutic intervention for FASD.

The use of ML in FASD diagnosis represents a significant advance in addressing the complexity and heterogeneity of this disorder. Traditional diagnostic methods for FASD are often hampered by factors such as the absence of maternal alcohol consumption confirmation, lack of characteristic facial dysmorphology, and subtle or absent growth impairment, all of which can lead to misdiagnosis or delayed intervention (Chasnoff et al., 2015), highlighting the need for novel diagnostic approaches to complement those already available. Early ML studies focused on predicting FASD risk in pregnant drinkers using questionnaire data assessing factors such as timing of alcohol consumption, race, ethnicity, prenatal care, and pregnancy complications (Oh et al., 2023). However, reliance on self-reported data introduces bias due to potential misrepresentation, emphasizing the limitations of such approaches. In contrast, biomarker-based analyses provide quantifiable, reproducible indicators of underlying pathophysiological processes, making them valuable tools for objective diagnosis.

To ensure the robustness of our approach, we selected biomarkers with strong evidence in the literature for being affected by PAE and that can be reliably quantified in serum using immunoassays. These biomarkers encompass key molecular pathways implicated in neuroinflammation and neurodevelopmental alterations induced by PAE. Specifically, cytokines and chemokines such as IFN-γ (Roberson et al., 2012), CX3CL1 (Bachstetter et al., 2011; Cardona et al., 2006; Pascual et al., 2017; Roberson et al., 2011), IL-1β (Milligan & Watkins, 2009; Pascual et al., 2017; Roberson et al., 2012), CCL2 (Chang et al., 2018; Pascual et al., 2017; Kai Zhang & Luo, 2019), IL-10 (Lobo-Silva et al., 2016; Milligan & Watkins, 2009), and CXCL16 (Juárez-Rodríguez et al., 2020; Lepore et al., 2018; Rosito et al., 2012, 2014) have been extensively associated with alcohol-induced immune dysregulation and neuroinflammation, produced by impaired activation of microglia and astrocytes (Kane & Drew, 2021; Lawrimore et al., 2019; Lord et al., 2022). Additionally, NGF-β, a critical mediator of neuronal plasticity and survival (Bersani et al., 2000), has been associated in the neurodevelopmental deficits observed in FASD (Boschen & Klintsova, 2017; Ceccanti et al., 2012).

The input features were deliberately limited to a subset of seven biomarkers that demonstrated statistically significant differences between groups and were strongly supported by prior biological evidence. This targeted feature selection aimed to reduce dimensionality and minimize the risk of overfitting, which is particularly important in studies with limited sample sizes (Ng et al., 2023). Including non-significant or weakly associated variables could increase model complexity and reduce generalizability by introducing noise into the predictive framework (Kannan, 2017). Furthermore, the use of a concise and informative biomarker panel improves clinical feasibility, decreases the burden of blood collection in pediatric populations, and enhances the potential for integration into real-world diagnostic workflows. By focusing on biomarkers that are both biologically relevant and statistically robust, the resulting machine learning models are designed to be accurate, interpretable, and suitable for practical clinical implementation.

Recent research has demonstrated the potential of ML algorithms in early FASD diagnosis, showing promising results by leveraging diverse datasets (Suttie et al., 2024). Ehrig et al. utilized physical traits (e.g., body length, head circumference) and neuropsychological measures (e.g., IQ, behavior, memory), achieving good levels of accuracy (0.85), precision (0.87), and sensitivity (0.91) (Ehrig et al., 2023). Goh et al. employed behavior scores, IQ data, and physical examinations to train a decision tree model, obtaining sensitivities of 64 - 81% and specificities of 78 - 80% (Goh et al., 2016). Zhang et al. introduced a comprehensive ML framework integrating eye movement metrics, psychometric tests, and brain imaging (Zhang et al., 2019). Other efforts have used advanced neuroimaging techniques, such as magnetic resonance imaging (Rodriguez et al., 2021) and diffusion tensor imaging combined with saccadic eye movements and executive function scores (Duarte et al., 2021). In addition, methylation signatures have been explored as biomarkers for FASD classification (Lussier et al., 2018). Fu et al. and Blanck-Lubarsch et al. used advanced facial recognition methodologies, employing facial datasets and automated classification of 3D facial scans (Blanck-Lubarsch et al., 2022; Fu et al., 2022). Ramos et al. developed ML models for FASD prediction based on sociodemographic, clinical, and neuropsychological data, demonstrating the utility of algorithmic approaches in this area (Ramos-Triguero et al., 2024).

In the present study, we take a novel approach by using blood-based biomarkers as predictive variables, being, to date, the first ML study to do so for FASD diagnosis. This study evaluated eight different ML algorithms, employing 5-fold cross-validation on the training set. Among them, RF model showed superior performance, achieving the highest accuracy (0.89), precision (0.92), sensitivity (0.92), F1 score (0.92), specificity (0.83), and ROC AUC (0.88), making it an effective model for predicting FASD diagnosis. The biomarkers identified as most influential in accurately predicting FASD were IL-10, IFNγ, CCL2, and NGFβ, have been well documented in previous studies for their role in FASD-related pathophysiology (Bodnar et al., 2016; Heberlein et al., 2013; Noor & Milligan, 2018).

The incorporation of serum biomarkers would not only enhance diagnostic accuracy but also provide valuable information on the underlying mechanisms of FASD. By utilizing these biomarkers, this approach addresses diagnostic challenges in complex cases without clear maternal alcohol history, facial dysmorphology, or growth impairment. A small, targeted panel of blood-based biomarkers offers a cost-effective and feasible diagnostic aid that could complement existing clinical criteria, improving early identification of children at risk. For future experiments, the use of blood-based diagnostics, combined with new technologies, such as wearable-based assessments, could refine FASD profiling, by capturing real-time physiological and behavioral data, offering a more comprehensive and non-invasive evaluation. This research underscored the importance of integrating biological markers with ML to increase diagnostic accuracy and improve clinical outcomes. Future studies should focus on refining these models, identifying additional biomarkers, and incorporating digital health tools to enhance diagnostic precision and clinical outcomes, ultimately improving the quality of life for individuals affected by FASD.

## Conclusions

This study highlights the utility of machine learning in advancing FASD diagnosis through the integration of serum biomarkers, addressing critical challenges posed by the heterogeneity of the disorder. Through the use of an RF model, we achieved robust predictive performance, with key biomarkers such as IL-10, IFNγ, CCL2, NGFβ, IL-1β, CX3CL1 and CXCL16, demonstrating significant associations with FASD pathophysiology. The novel use of blood biomarkers offers a non-invasive and objective diagnostic approach, especially valuable in cases where maternal alcohol consumption history is unavailable or traditional diagnostic features are absent. The inclusion of inflammatory and neurotrophic markers underscored the importance of immune and neurodevelopmental pathways in FASD, underscoring the urgent need for therapeutic interventions such as the use of EGCG. This study highlights the therapeutic potential of this natural antioxidant to mitigate FASD-associated inflammation and neurodevelopmental impairments. EGCG treatment was shown to significantly modulate key biomarkers such as IL-10, CX3CL1, IL-1β, IFNγ, and NGFβ, restoring levels associated with healthier physiological states.

## Limitations

This study provided valuable evidence of the potential of ML algorithms to help in the early diagnosis of FASD. Moreover, the analysis of key biomarkers demonstrated the potential benefits of EGCG treatment in FASD. However, it is important to note some limitations. The moderate sample size, partly influenced by the dropout rate related to behavioral and compliance challenges in children diagnosed with FASD, may have reduced the statistical power of our findings and affected the performance of the ML model. This provides an opportunity for future research in a larger and more diverse population to validate and expand our findings. Additionally, our study focused on specific biomarkers related to FASD in previous studies, not providing a complete picture of FASD profile. Exploring other potential biomarkers could offer a more comprehensive understanding of FASD. Furthermore, while our ML model showed promising results, additional work is needed to refine and test these models to assess their performance in clinical conditions. Importantly, adherence to the EGCG treatment posed a challenge due to the behavioral characteristics of children diagnosed with FASD, the burden of daily medication, and the procedural distress caused by repeated blood collections, all of which contributed to participant dropout. Lastly, while EGCG showed potential therapeutic effects, further research is needed to fully understand its mechanisms of action and long-term effects.

## Funding

This research was funded by 10.13039/501100004587Instituto de Salud Carlos III (ISCIII) and co-funded by the European Union (projects RD24/0013/0019 - Spanish network in maternal, neonatal, child and developmental health research RICORS-SAMID, PI19/01853, PI23/01220, PI21/01415) and 10.13039/501100003529European Union - NextGenerationEU/Mecanismo para la Recuperación y la Resiliencia (MRR)/ Plan de Recuperación, Transformación y Resiliencia (PRTR)(project RD21/0012/0017), Fundación Mutua Madrileña (ref. AP183662023), and with support of the Departament de Recerca i Universitats de la Generalitat de Catalunya al Grup de Recerca Infància i Entorn (GRIE) (2021 SGR 01290)." The image “Generalitat de Catalunya Departament de Recerca i Universitats” appears outside of its column and overlaps with some references. We would be grateful if you could adjust its placement to ensure proper alignment and readability.



## Ethics information

The study protocol was approved by Comité Ético de Investigación Clínica Parc de Salut MAR (No. HCB/2021/0459). All methods were performed in accordance with Declaration of Helsinki and Spanish guidelines and regulations for data privacy. Written consent was obtained from either the caregiver or the legal representative of the patient, as the patient was unable to provide informed consent on their own. This study is registered at www.clinicaltrials.gov (NCT02558933).

## CRediT authorship contribution statement

**Anna Ramos-Triguero:** Conceptualization, Writing – original draft, Writing – review & editing, Methodology, Formal analysis, Investigation. **Elisabet Navarro-Tapia:** Writing – original draft, Writing – review & editing, Methodology, Investigation, Conceptualization. **Melina Vieiros:** Conceptualization, Writing – original draft, Writing – review & editing, Methodology, Investigation. **Leopoldo Martínez:** Writing – review & editing, Methodology, Funding acquisition. **Óscar García-Algar:** Conceptualization, Writing – review & editing, Methodology, Funding acquisition. **Vicente Andreu-Fernández:** Conceptualization, Writing – original draft, Writing – review & editing, Methodology, Investigation, Supervision, Formal analysis.

## Declaration of competing interest

The authors declare that they have no known competing financial interests or personal relationships that could have appeared to influence the work reported in this paper.

## Appendix Supplementary materials

Image, application 1
