# Supplementary material for: Machine learning-driven blood biomarker profiling and EGCG intervention in fetal alcohol spectrum disorder
Source: Int J Clin Health Psychol. 2025 Sep 4;25(3):100620. doi: 10.1016/j.ijchp.2025.100620 (PMC12446210; doi:10.1016/j.ijchp.2025.100620)
Supplement: Supplementary file 1 [file mmc1.docx]

**Supplementary Tables**

**Supplementary Table 1.** Food supplement FontUp® composition

| **Average analysis** | **Per 100 mL** | **Per ration** |
| --- | --- | --- |
| **Energetic value (kJ/kcal)** | 1606/383 | 787/188 |
| **Fats (g)** | 11 | 5.4 |
| - **Saturated (g)** | 4.4 | 2.2 |
| - **Monounsaturated (g)** | 4.4 | 2.2 |
| - **Polyunsaturated (g)** | 2.2 | 1.1 |
| - **DHA (mg)** | 5.4 | 2.6 |
| **Carbohydrates (g)** | 47 | 23 |
| - **Dietary fibre (g)** | 16 | 7.9 |
| - **Fructo-ologosaccharides (g)** | 3.1 | 1.5 |
| **Proteins (g)** | 16 | 8 |
| **Salt (g)** | 0.52 | 0.3 |
| **Minerals** | | |
| **Na (mg)** | 206 | 101 |
| **K (mg)** | 606 | 297 |
| **Cl (mg)** | 395 | 194 |
| **Ca (mg)** | 221 | 108 |
| **P (mg)** | 209 | 102 |
| **Mg (mg)** | 106 | 52 |
| **Fe (mg)** | 4.6 | 2.3 |
| **Zn (mg)** | 3.1 | 1.5 |
| **Cu (μg)** | 0.5 | 0.2 |
| **Mn (mg)** | 0.5 | 0.2 |
| **F (μg)** | 0.1 | 0.05 |
| **Se (μg)** | 10 | 4.9 |
| **Cr (μg)** | 16 | 7.8 |
| **Mo (μg)** | 10 | 4.9 |
| **I (μg)** | 21 | 10 |
| **Vitamins** | | |
| **Vitamin A (μg)** | 251 | 123 |
| **Vitamin D (μg)** | 1.9 | 0.9 |
| **Vitamin E (mg)** | 4.2 | 2.1 |
| **Vitamin K (μg)** | 15.0 | 7.4 |
| **Vitamin C (mg)** | 25 | 12 |
| **Thiamine (B1) (mg)** | 0.3 | 0.1 |
| **Riboflavin (B2) (mg)** | 0.4 | 0.2 |
| **Niacin (B3/PP) (mg)** | 4.0 | 2.0 |
| **Vitamin B6 (mg)** | 0.4 | 0.2 |
| **Folic acid (B9) (μg)** | 80.0 | 39 |
| **Vitamin B12 (μg)** | 0.5 | 0.2 |
| **Biotin (μg)** | 58.0 | 28 |
| **Pantothenic acid (B5) (mg)** | 1.9 | 0.9 |
| **Others** | | |
| **Green tea extract (mg) (minimum 250 mg EGCG per ration)** | 543 | 266 |
| **Osmolarity (mOsm/L)** | 635 | |

**Supplementary Table 2.** Primer sequences used for real-time quantitative reverse transcriptase polymerase chain reaction (RT-qPCR).

| **Gene** | **5' to 3'** | **Sequence** |
| --- | --- | --- |
| *CCL2* | Sense | CCCCAGTCACCTGCTGTTAT |
| *CCL2* | Anti-Sense | AGATCTCCTTGGCCACAATG |
| *CX3CL1* | Sense | TGTGACGAAATGCAACATCA |
| *CX3CL1* | Anti-Sense | TGTGCTGTCTCGTCTCCAAG |
| *CXCL16* | Sense | GAGGTTCCAGCTCCTTTCCT |
| *CXCL16* | Anti-Sense | CACAATCCCCGAGTAAGCAT |
| *IL-1β* | Sense | GGACAAGCTGAGGAAGATGC |
| *IL-1β* | Anti-Sense | TCGTTATCCCATGTGTCGAA |
| *IL-10* | Sense | TTACCTGGAGGAGGTGATGC |
| *IL-10* | Anti-Sense | GGCCTTGCTCTTGTTTTCAC |
| *IL-6* | Sense | CACACAGACAGCCACTCACC |
| *IL-6* | Anti-Sense | TTTTCTGCCAGTGCCTCTTT |
| *VCAM-1* | Sense | AAGATGGTCGTGATCCTTGG |
| *VCAM-1* | Anti-Sense | GGTGCTGCAAGTCAATGAGA |
| *MMP-10* | Sense | GTGGAGTTCCTGACGTTGGT |
| *MMP-10* | Anti-Sense | TCAATGGCAGAATCAACAGC |
| *IFNγ* | Sense | TGACCAGAGCATCCAAAAGA |
| *IFNγ* | Anti-Sense | CATGTATTGCTTTGCGTTGG |
| *Enolase-2* | Sense | GGTCCAAGTTCACAGCCAAT |
| *Enolase-2* | Anti-Sense | CAGTTGCAGGCCTTTTCTTC |
| *NGF**β* | Sense | CAACAGGACTCACAGGAGCA |
| *NGFβ* | Anti-Sense | GTCTGTGGCGGTGGTCTTAT |
| *S100β* | Sense | ATTCTGGAAGGGAGGGAGAC |
| *S100β* | Anti-Sense | TCCACAACCTCCTGCTCTTT |
| *GAPDH* | Sense | TGCACCACCAACTGCTTAGC |
| *GAPDH* | Anti-Sense | GGCATGGACTGTGGTCATGAG |

**Supplementary Table 3.** Serum levels of cytokines, chemokines, growth factors and neural biomarkers according to groups.

|  | Control | FASD | FASD + EGCG | Adj p-value; Multiple comparisons | | |
| --- | --- | --- | --- | --- | --- | --- |
|  |  |  |  | Ctrl vs FASD | Ctrl vs EGCG | FASD vs FASD+EGCG |
| IFNy | 7.1 (3.2) | 11.3 (6.2) | 6.7 (4.2) | **0.041** | 0.999 | **0.006** |
| CX3CL1 | 1,104 (291.5) | 1,343 (465.2) | 953.5 (287.5) | **0.043** | 0.2 | **<0.001** |
| CCL2 | 211.5 (54.1) | 255.7 (77.35) | 225.9 (75.0) | **0.031** | 0.463 | 0.144 |
| IL-1β | 9.3 (1.2) | 11.0 (2.1) | 9.4 (1.9) | **<0.001** | 0.999 | **0.018** |
| IL-10 | 4.0 (1.8) | 2.7 (1.5) | 4.5 (2.7) | **0.036** | 0.999 | **0.016** |
| CXCL16 | 2,095 (273.4) | 1,921 (252.8) | 1926 (334.5) | **0.031** | 0.053 | 0.945 |
| IL-6 | 0.6 (0.5) | 0.7 ± 0.5) | 0.5 (0.4) | 0.999 | 0.491 | 0.202 |
| VCAM-1 | 1,297,620 (632,391) | 1,509,900 (953,547) | 1,102,314 (527,170) | 0.999 | 0.815 | 0.219 |
| MMP-10 | 691.9 (384.0) | 680.4 (369.1) | 613.2 (332.7) | 0.999 | 0.999 | 0.999 |
| NGFβ | 3.3 (1.2) | 2.6 (0.7) | 3.2 (1.1) | **0.004** | 0.513 | **0.029** |
| Enolase 2 | 8,874 (4130) | 11,156 (4,804) | 9,567 (3527) | 0.232 | 0.999 | 0.766 |
| S100β | 80.6 (38.5) | 72.2 (49.7) | 70.2 (47.9) | 0.907 | 0.843 | 0.999 |

Biomarker concentrations (pg/mL) are represented by mean and standard deviation (SD). Abbreviations: CCL2, C-C Motif Chemokine Ligand 2; Ctrl, Control; CX3CL1, C-X3-C Motif Chemokine Ligand 1; CXCL16, C-X-C Motif Chemokine Ligand 16; FASD, Fetal Alcohol Spectrum Disorders; IL-1β, Interleukin 1 Beta; IL-10, Interleukin 10; IL-6, Interleukin 6; IFNγ, Interferon Gamma; VCAM-1, Vascular Cell Adhesion Molecule 1; MMP-10, Matrix Metallopeptidase 10; NGFβ, Nerve Growth Factor Beta; s100β, S100 Calcium Binding Protein B. Bold indicates statistical significance.

**Supplementary Table 4.** **Pairwise comparisons of X centroid positions across control, FASD, and EGCG groups.** The table summarizes the results of pairwise t-tests with pooled standard deviation, assessing differences in the X centroid coordinate between groups. Bold indicates significant bonferroni-adjusted p-values.

|  | FASD | EGCG |
| --- | --- | --- |
| Control | 0.34 | 1.00 |
| FASD | 1.00 | **0.03** |

**Supplementary Table 5.** **Pairwise comparisons of Y centroid positions across control, FASD, and EGCG groups.** The table summarizes the results of pairwise t-tests with pooled standard deviation, assessing differences in the Y centroid coordinate between groups. Bold indicates significant bonferroni-adjusted p-values.

|  | FASD | EGCG |
| --- | --- | --- |
| Control | **0.007** | 1.000 |
| FASD | 1.000 | **0.016** |

**Supplementary Table 6.** **Pairwise comparisons of Z centroid positions across control, FASD, and EGCG groups.** The table summarizes the results of pairwise t-tests with pooled standard deviation, assessing differences in the Z centroid coordinate between groups. Bold indicates significant bonferroni-adjusted p-values.

|  | FASD | EGCG |
| --- | --- | --- |
| Control | 0.850 | 0.170 |
| FASD | 1.000 | 0.770 |

**Supplementary Table 7**. **Probabilities of Random Forest Model Predictions for control, FASD, and EGCG Groups**. The table presents the predicted probabilities assigned by the Random Forest model to each sample, indicating its classification confidence for Control and FASD.

|  | Control | FASD |
| --- | --- | --- |
| 143e | 0.768 | 0.232 |
| 149e | 0.032 | 0.968 |
| 198e | 0.65 | 0.35 |
| 146e | 0.282 | 0.718 |
| 169e | 0.708 | 0.292 |
| 109e | 0.73 | 0.27 |
| 168e | 0.35 | 0.65 |
| 226e | 0.094 | 0.906 |
| 221e | 0.026 | 0.974 |
| 135e | 0.12 | 0.88 |
| 174e | 0.092 | 0.908 |
| 175e | 0.072 | 0.928 |
| 189e | 0.584 | 0.416 |
| 159e | 0.578 | 0.422 |
| 164e | 0.718 | 0.282 |
| 155e | 0.864 | 0.136 |
| U179e | 0.42 | 0.58 |
| M28e | 0.202 | 0.798 |
| M12e | 0.11 | 0.89 |
| M4e | 0.848 | 0.152 |
| M27e | 0.52 | 0.48 |
| M20e | 0.026 | 0.974 |
| U226e | 0.774 | 0.226 |
| U256e | 0.42 | 0.58 |
| U136e | 0.076 | 0.924 |
| U212e | 0.248 | 0.752 |
| M10e | 0.136 | 0.864 |
| U101e | 0.144 | 0.856 |
| M16e | 0.09 | 0.91 |
| M15e | 0.076 | 0.924 |
